# Cardiovascular Risk in Women With Nonclassical Congenital Adrenal Hyperplasia

**DOI:** 10.1210/clinem/dgae155

**Published:** 2024-03-11

**Authors:** Fernanda Cavalieri Costa, Larissa Garcia Gomes, Thais Martins de Lima, Luiz Aparecido Bortolotto, Valeria Hong, Renata Verardino, Manoel de Souza Rocha, Serli Kiyomi Nakao Ueda, Mirela Costa de Miranda, Heraldo Possolo de Souza, Ana Claudia Latronico, Berenice Bilharinho Mendonca, Tania A S S Bachega

**Affiliations:** Laboratório de Hormônios e Genética Molecular- LIM/42, Departamento de Clínica Médica, Disciplina de Endocrinologia e Metabologia, Unidade de Adrenal, Hospital das Clínicas, Faculdade de Medicina da Universidade de São Paulo, 05403-000 São Paulo, Brazil; Laboratório de Hormônios e Genética Molecular- LIM/42, Departamento de Clínica Médica, Disciplina de Endocrinologia e Metabologia, Unidade de Adrenal, Hospital das Clínicas, Faculdade de Medicina da Universidade de São Paulo, 05403-000 São Paulo, Brazil; Laboratório de Emergências Clínicas (LIM 51), Hospital das Clínicas, Faculdade de Medicina da Universidade de São Paulo, 05403-000 São Paulo, Brazil; Instituto do Coração (INCOR), Hospital das Clínicas da Faculdade de Medicina da Universidade de São Paulo, 05403-000 São Paulo, Brasil; Instituto do Coração (INCOR), Hospital das Clínicas da Faculdade de Medicina da Universidade de São Paulo, 05403-000 São Paulo, Brasil; Instituto do Coração (INCOR), Hospital das Clínicas da Faculdade de Medicina da Universidade de São Paulo, 05403-000 São Paulo, Brasil; Instituto de Radiologia, Hospital das Clínicas da Faculdade de Medicina da Universidade de São Paulo, 05403-000 São Paulo, Brazil; Instituto de Radiologia, Hospital das Clínicas da Faculdade de Medicina da Universidade de São Paulo, 05403-000 São Paulo, Brazil; Laboratório de Hormônios e Genética Molecular- LIM/42, Departamento de Clínica Médica, Disciplina de Endocrinologia e Metabologia, Unidade de Adrenal, Hospital das Clínicas, Faculdade de Medicina da Universidade de São Paulo, 05403-000 São Paulo, Brazil; Laboratório de Emergências Clínicas (LIM 51), Hospital das Clínicas, Faculdade de Medicina da Universidade de São Paulo, 05403-000 São Paulo, Brazil; Laboratório de Hormônios e Genética Molecular- LIM/42, Departamento de Clínica Médica, Disciplina de Endocrinologia e Metabologia, Unidade de Adrenal, Hospital das Clínicas, Faculdade de Medicina da Universidade de São Paulo, 05403-000 São Paulo, Brazil; Laboratório de Hormônios e Genética Molecular- LIM/42, Departamento de Clínica Médica, Disciplina de Endocrinologia e Metabologia, Unidade de Adrenal, Hospital das Clínicas, Faculdade de Medicina da Universidade de São Paulo, 05403-000 São Paulo, Brazil; Laboratório de Hormônios e Genética Molecular- LIM/42, Departamento de Clínica Médica, Disciplina de Endocrinologia e Metabologia, Unidade de Adrenal, Hospital das Clínicas, Faculdade de Medicina da Universidade de São Paulo, 05403-000 São Paulo, Brazil

**Keywords:** nonclassical congenital adrenal hyperplasia, cardiovascular risk, visceral fat, pulse wave velocity, endothelial function, carotid intima media thickness

## Abstract

**Context:**

The outcomes related to cardiovascular risk (CVR) in patients with the nonclassical form of congenital adrenal hyperplasia (NCAH) are unknown, especially those related to therapeutic options, including low doses of glucocorticoids or oral contraceptive pills.

**Objectives:**

To analyze CVR by markers of atherosclerosis in females with the nonclassical form according to therapeutic options.

**Design and Setting:**

A cross-sectional study at a tertiary center.

**Patients and Methods:**

Forty-seven females with NCAH (33.4 ± 10 years) were subdivided into group 1 (G1) (n = 28) treated with dexamethasone (0.14 ± 0.05 mg/m^2^/day), group 2 (G2) (n = 19) treated with oral contraceptive pills, and group 3 (G3) (30 matched controls). CVR was analyzed through serum lipids, the Homeostatic Model Assessment for Insulin Resistance (HOMA-IR), inflammatory cytokines levels, and quantitative image evaluations [pulse wave velocity (PWV), endothelial function by flow-mediated dilatation (FMD), carotid intima media thickness (CIMT), and visceral fat (VAT) by abdominal tomography].

**Results:**

There were no statistically significant differences in BMI, HOMA-IR, high-density lipoprotein-cholesterol, or triglyceride levels among groups (*P* > .05). Serum interleukin 6 (IL-6) levels were higher in G1 than in G2 (*P* = .048), and interleukin 8 (IL-8) levels were higher in G1 than in G2/3 (*P* = .008). There were no statistically significant differences in VAT, PWV, FMD, or CIMT among groups (*P* > .05). In multivariable regression analysis, there was no statistically significant association between glucocorticoid dose and evaluated outcomes.

**Conclusion:**

Adult females with NCAH did not show increased CVR using methodologies for detection of precocious atherosclerosis. Although patients receiving dexamethasone therapy had increased IL-6 and IL-8 levels, these data were not associated with radiological markers of atherosclerosis. Our cohort was composed of young adults and should be reevaluated in a long-term follow-up.

The nonclassical form of congenital adrenal hyperplasia (CAH) is a common autosomal recessive disease that affects up to 1% of the general population. It is caused by mutations in the *CYP21A2* gene that impair 21-hydroxylase activity, resulting in decreased cortisol production and increased secretion of adrenal androgens (1-3). Clinical manifestations in adult females include menstrual abnormalities, hirsutism, acne, and/or infertility, which are similar to those of polycystic ovarian syndrome (PCOS) (1, 4). Traditionally, children with NCAH are treated with short-life glucocorticoids to preserve potential final height (1). However, there is no consensus regarding the optimal therapy for adult females; some centers use low glucocorticoid (GC) doses to reduce adrenal hyperandrogenism, while others use symptom-directed medications such as oral contraceptive pills (OCPs) to normalize the menstrual cycle and/or cyproterone acetate or spironolactone for hirsutism (1, 5-8).

It has been hypothesized that patients with CAH could have a higher risk of metabolic syndrome than the general population due to chronic androgen exposure, as observed in PCOS patients (4). Previous studies involving adult and pediatric patients with classical forms of CAH, the most severe clinical form, evidenced a higher prevalence of obesity, dyslipidemia, and insulin resistance (9-14). Data about the prevalence of metabolic syndrome components as well as visceral adiposity in adult patients with NCAH treated with or without GCs are not available so far.

Cardiovascular risk (CVR) can be influenced by chronic hyperandrogenism as well as chronic hypercortisolism. Williams et al (14) evaluated body composition and insulin resistance in 37 children with CAH (26 with classical forms, 11 with the nonclassical form; median age 9.4 years) and compared them with 41 matched controls. Higher fat mass was identified in children with classical forms, and, interestingly, insulin resistance was detected in patients with NCAH in comparison with controls, although they had a lower percentage of fat mass than those with the classical forms. These findings suggested that postnatal hyperandrogenism has adverse metabolic effects on insulin sensitivity, since children with NCAH are diagnosed, on average, 5 years later than those with classical forms. This theory can be supported by a Chinese study comprising 30 newly diagnosed women with the simple virilizing form of CAH; they were nonobese, and, despite the absence of GC therapy, they showed reduced insulin sensitivity compared to controls (15). Another cohort comprising 14 premenopausal women with NCAH who had never received GC therapy presented a higher frequency of insulin resistance in comparison with 20 matched controls (16). Women with PCOS exposed to increased androgen levels showed increased visceral adipose tissue (VAT) in relation to controls, and a positive correlation with serum testosterone levels was observed (17). These data may explain the higher incidence of insulin resistance and dyslipidemia in PCOS patients, probably secondary to androgen exposure (4, 17, 18).

CVR has been evaluated by structural parameters such as carotid intima-media thickness (CIMT) and, more recently, by the following functional parameters: (1) aortic pulse wave velocity (PWV), which quantifies arterial stiffness, and (2) endothelial function, which is observed through flow-mediated dilatation (FMD) in the brachial artery. Functional parameters are early markers of CVR and can precede the development of atherosclerosis even before the identification of arterial structural changes (19-21). A few studies have evaluated these parameters in patients with classical forms, and they included patients receiving mixed GC regimens (22-25). Increased CIMT was observed in young patients in relation to matched controls and was positively correlated with serum androgen levels (22-24). On the other hand, in an Australian cohort including 14 adolescents with classical forms of CAH, the CIMT was normal, but impaired vascular endothelial function through FMD was identified in comparison with controls (25). Another cohort of 19 young Polish women with classical forms also presented lower FMD compared to matched controls (26).

As there is no study in the literature evaluating cardiovascular risk (CVR) in patients with NCAH, our objective was to evaluate whether adult patients have increased CVR. Subsequently, we aimed to evaluate whether this risk was associated with long-term GC therapy or chronic hyperandrogenism exposure. Answering this question will be useful to define the optimal therapy for patients with the nonclassical form, which is still controversial.

##  

### Patients

The study was approved by the Ethical Committee of Hospital das Clínicas da Faculdade de Medicina da Universidade de São Paulo (Process #70167417.2.0000.0068), and written consent was obtained from all patients.

The cohort included 47 women, with a mean age of 33.4 ± 10 years and a hormonal diagnosis of NCAH characterized by basal/ACTH-stimulated 17OH-progesterone (17OHP) levels >10 ng/mL (1). Since ACTH-stimulated 17OHP levels between 10 and 14 ng/mL could comprise NCAH and heterozygotes subjects, for these patients, *CYP21A2* molecular analysis was also performed to confirm the NCAH diagnosis, as previously described (3).

Forty-seven NCAH females with a mean age of 33.4 ± 9 years were enrolled; 14/47 patients were diagnosed during infancy due to precocious pubarche, and the remaining 33 were diagnosed during adolescence/adulthood due to hyperandrogenic manifestations and/or menstrual abnormalities. Patients diagnosed in childhood received cortisone acetate during growth periods; after reaching the final height, the glucocorticoid (GC) was changed by dexamethasone; most of these patients were comfortable maintaining the GC treatment during adulthood, since this therapy allows regular menstrual cycles and ameliorates the hirsutism (7). Among all 47 patients, 28 (59.6%) received dexamethasone during adulthood (group 1); the mean dose was 0.14 ± 0.05 mg/m^2^/day, and the mean duration of therapy was 10.5 ± 7 years. On the other hand, many patients diagnosed in adulthood preferred the use of OCPs and antiandrogenic drugs. The remaining 19 (40.4%) females received OCPs alone or in association with spironolactone (varying from 100 to 300 mg/day) (group 2). The mean treatment time was 5.3 ± 5 years. Twenty-one percent of patients used ethinyl estradiol (EE) + gestodene, 37% EE + drospirenone, 32% EE + cyproterone acetate, 5% medroxyprogesterone, and 5% a levonorgestrel-releasing intrauterine device. Patients in group 2 had never received GC replacement therapy.

Thirty controls, with a mean age of 37.4 ± 9 years, without hyperandrogenic manifestations and with normal menstrual cycles, were also evaluated (group 3). The controls were matched for age and body mass index (BMI). These healthy controls were selected from the general population, respecting the exclusion criteria, which were pregnancy, smoking, and other associated comorbidities. The ethnicity of patients and controls was mixed, largely descended from Europeans, indigenous, Amerindian, and African people. European ancestry is predominant in all regions of Brazil.

## Methods

CVR was evaluated through BMI, serum concentrations of lipids, the Homeostatic Model Assessment for Insulin Resistance (HOMA-IR), and inflammatory cytokines and quantitative image evaluations, including CIMT, aortic PWV, endothelial function by FMD, and quantification of visceral fat by abdominal computed tomography.

### Biochemical and Hormonal Assays

Glucose levels were determined by an automatic enzymatic colorimetric method using hexokinase (Cobas Integra, Roche, Basel, Switzerland), and insulin levels were determined by the Auto Delfia fluoroimmunoassay (PerkinElmer, Turku, Finland). Total cholesterol, high-density lipoprotein cholesterol (HDL-c), and triglyceride (TG) levels were analyzed by an automatic enzymatic colorimetric method (Cobas Mira, F. Hoffmann-La Roche, Basel, Switzerland) with commercial kits from Roche (Mannheim, Germany). Insulin resistance was assessed by the HOMA-IR using the following formula: insulin resistance = insulin (μU/mL) × glucose (mmol/L)/22.5. Serum analysis of 17OHP, androstenedione, and cortisol levels was performed by liquid chromatography–mass spectrometry, and total testosterone levels were measured by electrochemiluminescence (Cobas e602 Roche Diagnostics, Indianapolis, IN, USA). The functional sensitivity of the method was 12 ng/mL. The intra- and interassay coefficients of variation were 7.1% and less than 15%, respectively.

Mean daily doses of cortisone acetate (used during growth periods) and dexamethasone (after its introduction in adulthood) were determined according to body surface; the cumulative dose was calculated by multiplying the mean daily dose per body surface by the number of days that GCs were used. Dexamethasone doses were converted to hydrocortisone equivalents considering the following ratio and expressed in mg/m^2^: 30 mg hydrocortisone = 37.5 cortisone acetate = 0.75 dexamethasone.

### Endothelial Function

Patients were instructed not to drink alcoholic beverages or consume products containing caffeine/chocolate 1 day before the test. Endothelial function was evaluated in the morning after an 8-hour fast by the same examiner. FMD was assessed sequentially in the right brachial artery. The artery diameter was verified by ultrasonography (Sequoia Echocardiography System, version 6.0, Acuson, Siemens, CA, USA) equipped with a high-resolution linear transducer (7-12 MHz) and coupled to a computer. In the reactive hyperemia phase (FMD), the sphygmomanometer was inflated 50 mmHg above the systolic blood pressure for 5 minutes. Then, the blood flow was restored, and the diameter of the brachial artery after hyperemia was compared with the basal diameter and was expressed as a percentage of variation. The degree of vasodilation obtained was expressed as a percentage in relation to basal values (27).

### Aortic Pulse Wave Velocity

The carotid-femoral pulse wave velocity was measured by the same observer using an automatic device (Complior SP, Artech Medical, Pantin, France). A pressure sensor was placed on the right carotid artery, and another was placed on the right femoral artery in the supine position. Each wave appeared in real time on the computer screen, and the device determined the start of the wave at both locations and the PWV. Two measurements were obtained for each participant. The PWV values were standardized to the “real” carotid–femoral distance by multiplying by 0.8 (28).

### Carotid Intima Thickness

Two measurements of CIMT were assessed on the opposite wall of both common carotid arteries. Fixed images were analyzed using edge detection software (Prowin™) (29).

### Subcutaneous and Visceral Abdominal Fat

The evaluation of subcutaneous and visceral abdominal fat was performed by computed tomography using multidetectors, 2 with 64 channels (Philips Medical Systems and GE) and 1 with 128 channels (Toshiba), with the following parameters: 120 kVp and mA variable according to the regulation system dose of each appliance. Initial images were performed to locate the L4-L5 disc space. Subsequently, a sequence of 7 images was obtained, sent to an AW VolumeShare 4 (GE) workstation, and processed by a fat attenuation voxel measurement system (range of −150 UH to −50 UH) after manual exclusion of the intestinal loops. Subcutaneous fat was considered the region situated between the skin and the external surface of the rectus abdominis muscle, external oblique, and muscles of the dorsal region. Visceral fat was considered to surround the straight abdominal muscles, external obliques, lumbar square, psoas muscles, and lumbar spine.

### Statistical Analysis

Descriptive statistics were presented as absolute and relative frequencies for qualitative variables and as the means with standard deviations and medians with interquartile ranges (IQR) for quantitative variables. The comparison of the main quantitative variables in relation to the group variable (group 1, group 2, and group 3) was carried out using the nonparametric Kruskal–Wallis test, followed by post hoc Dunn's test (when the Kruskal–Wallis test was rejected) for multiple comparisons with the Holm correction method to adjust for the familywise error rate. The normality assumption was evaluated using the Shapiro–Francia test and a Quantile–Quantile plot, and the homogeneity of variances was checked using the Levene test. Bivariate correlations between the quantitative variables were computed using Spearman's rank correlation coefficient. The main analyses were performed by fitting univariable and multivariable generalized linear models considering VAT, VAT/subcutaneous fat ratio (VAT/SAT), FMD, PWV, CIMT, and IL-6 and IL-8 levels as outcomes and age, BMI, androstenedione, testosterone, HDL-c, and TG levels and HOMA-IR as covariates. The gamma distribution with log-link function was utilized to identify the factors associated with the outcomes (VAT, VAT/SAT, PWV, IL-6, and IL-8 levels), while the normal distribution with the identity link function was considered to model FMD and CIMT as a function of the covariates in the generalized linear models. All hypothesis tests were 2-sided at a 5% significance level. Thus, results with *P*-values lower than .05 were considered statistically significant. All statistical analyses were conducted using statistical software R version 4.1 (R Core Team, 2020).

## Results

Twenty-eight patients in group 1 received dexamethasone during adulthood at a mean dose of 0.14 ± 0.05 mg/m^2^/day. Fourteen out of 28 patients were diagnosed during childhood due to precocious pubarche and received cortisone acetate at a mean dose of 16 ± 5 mg/m^2^/day for 5.9 ± 1.9 years, until the achievement of final height; after that, dexamethasone therapy was initiated. Nineteen patients in group 2 received OCPs alone or in combination with spironolactone at a dose varying between 100 and 300 mg/day.

The mean ages of groups 1, 2, and 3 were 33.1 ± 9 years, 33.8 ± 11 years, and 37.4 ± 9 years, respectively (*P* > .05); the mean BMIs were 29.4 ± 6.6 kg/m^2^, 26.3 ± 6.1 kg/m^2^, and 28.3 ± 5.6 kg/m^2^, respectively (*P* > .05) (Table 1).

**Table 1.** Anthropometric characteristics, metabolic profile, and serum hormonal levels of patients with nonclassical form treated with glucocorticoid (group 1), symptomatically (group 2), and matched controls (group 3)

	Group 1 (n = 28)	Group 2 (n = 19)	Group 3 (n = 30)	*P-*value
Age (years)	33.1 ± 9.1	33.8 ± 11	37.4 ± 9	.164
BMI (Kg/m^2^)	29.4 ± 6.6	26.3 ± 6.1	28.3 ± 5.6	.240
HOMA IR	5.1 ± 9.9	2.3 ± 1.6	2.6 ± 1.6	.079
HDL (mg/dL)	57.4 ± 17.6	56.3 ± 17.1	61.8 ± 19.7	.493
Triglycerides (mg/dL)	101.9 ± 39.2	98.9 ± 47.2	91.4 ± 38.8	.506
Testosterone (ng/dL)	43.9 ± 17.8*	51.3 ± 36.5*	23.2 ± 14.4	<.001
Androstenedione(ng/mL)	1.85 ± 0.6*	1.89 ± 1.0*	0.66 ± 0.2	<.001
17OHP (ng/mL)	6.9 ± 4.4*	7.3 ± 4.5*	0.5 ± 0.1	<.001
Leptin (pg/mL)	7885 (2925-10093)	2955 (1653-5869)	4987 (2539-6996)	.069
TNF-α (pg/mL)	1.62 (1.06-2.71)	2.33 (1.19-2.78)	1.86 (1.08-2.8)	.989
IL-6 (pg/mL)	1.65 (0.84-5.45)*	0.82 (0.38-1.18)*	0.91 (0.47-3.9)	.048
IL-8 (pg/mL)	1.75 (0.51-8.86)*	0.66 (0.09-1.37)*	0.63 (0.08-1.39)*	.008
PAI-1 (pg/mL)	16517 (11286-20249)	17509 (9330-19927)	13746 (8256-18979)	.684
VAT (cm^2^)	110.9 ± 59.3	94 ± 58.5	97.1 ± 43.2	.452
VAT/SAT	0.27 ± 0.1	0.30 ± 0.1	0.25 ± 0.1	.326
PWV (m/s)	6.8 ± 0.6	6.71 ± 0.6	7 ± 0.8	.436
FDM (%)	4.61 ± 3.27	6.41 ± 4.47	6.12 ± 3.45	.251
R CIMT (mm)	0.50 ± 0.06	0.47 ± 0.07	0.50 ± 0.06	.086
L CIMT (mm)	0.54 ± 0.08	0.52 ± 0.09	0.53 ± 0.07	.657

Serum testosterone, 17OHP, and androstenedione levels represent average over the last 5 years patients’ values.

Abbreviations: 17OHP, 17-hydroxyprogesterone; BMI, body mass index; FMD, flow-mediated dilation; HDL, high-density lipoprotein; HOMA IR, Homeostatic Model Assessment for Insulin Resistance; IL-6, interleukin 6; IL-8, interleukin 8; L CIMT, left carotid intima-media thickness; PAI-1, plasminogen activating factor; PWV, pulse wave velocity; R CIMT, right carotid intima-media thickness; TNF-α, TNF alpha; VAT, subcutaneous visceral fat; VAT/SAT, visceral fat/subcutaneous fat ratio.

^*^ *P* < .05.

There were no statistically significant differences in serum low-density lipoprotein cholesterol, HDL-c, and TG values among the groups, and HOMA-IR values were also similar (*P* > .05). All patients and controls presented normal blood pressure values. Serum testosterone and androstenedione levels were higher in groups 1 and 2 than in group 3 but were similar between groups 1 and 2 (*P* > .05) (Table 1).

**Figure 1.**
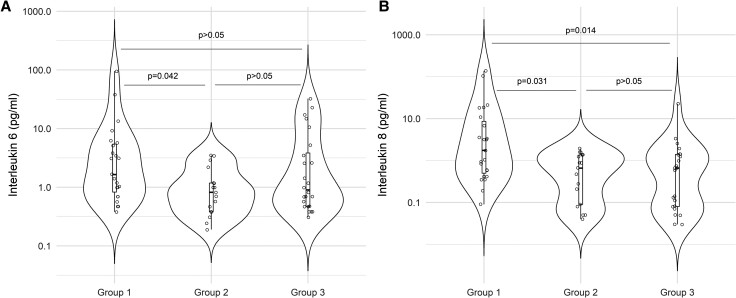
Serum IL-6 and IL-8 levels (pg/mL) among patients with nonclassical form of congenital adrenal hyperplasia treated with low doses of glucocorticoid (group 1), treated symptomatically (group 2), and matched controls (group 3). (A) IL-6 levels and (B) IL-8 levels. Abbreviations: IL-6, interleukin 6; IL-8, interleukin 8.

There was a statistically significant difference in serum IL-6 levels among the 3 groups (1.65 IQR 0.84-5.45 in group 1; 0.82 IQR 0.38-1.18 in group 2; and 0.91 IQR 0.47-3.9 in group 3, *P* = .048). In the 2 × 2 analysis, higher serum IL-6 values were observed in group 1 compared to group 2 (*P* = .042), but there was no statistically significant difference in serum IL-6 levels between group 1 vs the control group or between group 2 vs the control group (*P* > .05) (Fig. 1).

**Figure 2.**
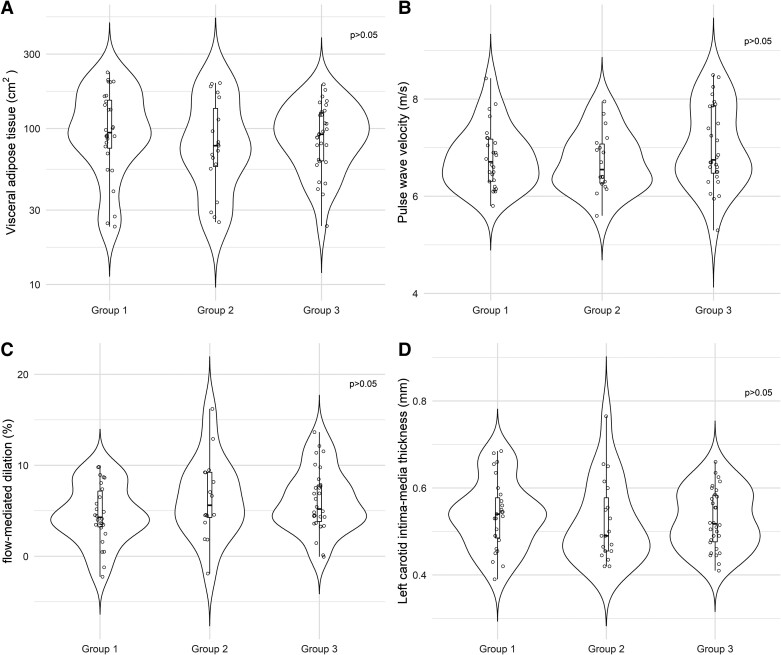
Comparison of visceral adipose tissue (A), pulse wave velocity (B), flow mediated dilatation (C), and left carotid intima-media thickness (D) among patients with nonclassical form of congenital adrenal hyperplasia. Group 1 patients treated with low doses of dexamethasone, group 2 patients treated with oral contraceptive pills and/or spironolactone, and group 3 matched controls.

Serum IL-8 levels were higher in group 1 than in the other groups (1.75 IQR 0.51-8.86 in group 1; 0.66 IQR 0.09-1.37 in group 2, and 0.63 IQR 0.08-1.39 in group 3, *P* = .008) (Fig. 2). In 2 × 2 comparisons, this difference remained statistically significant between group 1 vs the control group (*P* = .014) and group 1 vs group 2 (*P* = .031), but there was no significant difference between group 2 vs the control group (*P* > .05). There was no correlation between serum IL-6/IL-8 and androgen levels in groups 1 and 2 or between the duration of therapy or cumulative dose of GCs in group 1 (*P* > .05). There was no statistically significant difference in serum TNF-alpha, leptin, or plasminogen activating factor (PAI-1) levels among the groups (*P* > .05) (Table 1).

There was no statistically significant difference in the VAT/SAT ratio among groups 1, 2, and 3 (0.27 ± 0.1, 0.3 ± 0.1, 0.25 ± 0.1, respectively, *P* > 0.05) or in 2 × 2 comparisons (*P* > .05) (Table 1). There was a statistically significant positive correlation between the VAT area and HOMA-IR value in group 1 (r = 0.621, *P* < .0001), group 2 (r = 0.730, *P* < .001), and the control group (r = 0.464, *P* = .01). A statistically significant positive correlation was also observed between VAT area and leptin levels in groups 1 and 2 (group 1: r = 0.701, *P* < .0001; group 2: r = 0.539, *P* = .003). In group 2, there was a statistically significant positive correlation between VAT area and serum 17OHP levels (r = 0.481, *P* = .037) and a statistically significant negative correlation of VAT area with serum HDL-c values (r = −0.6, *P* = .003).

There was no statistically significant difference in PWV among groups 1, 2, and 3 (6.8 ± 0.6, 6.71 ± 0.6, 7 ± 0.8 m/s, respectively, *P* = 0 > .05) (Table 1). In groups 1 and 3, a statistically significant positive correlation between PWV and age was observed (group 1: r = 0.684, *P* = .001; group 3: r = 0.806, *P* < .0001). In group 1, there was a weak positive correlation between PWV and the duration of GC therapy (r = 0.444, *P* = .02), which was not observed in the multivariable regression analysis.

FMD was similar among the groups (4.6 ± 3.2%, 6.41 ± 4.4%, 6.12 ± 3.45% in groups 1, 2, and 3, respectively; *P* > .05) (Table 1). In group 2, there was a statistically significant negative correlation between FMD and BMI (r = −0.556, *P* = .025).

There was no statistically significant difference in CIMT among the groups (0.54 ± 0.08 mm, 0.52 ± 0.09 mm, 0.53 ± 0.07 mm for left CIMT in groups 1, 2, and 3, respectively; *P* > .05) (Table 1). There was a statistically significant positive correlation between the left CIMT and age in groups 1 and 3 (group 1: r = 0.403, *P* = 0.037; group 3: r = 0.544, *P* = .002).

There was no statistically significant correlation between VAT, VAT/SAT ratio, PWV, FMD, CIMT, and serum levels of testosterone, androstenedione, or interleukins in groups 1 and 2 or between the cumulative dose of GCs and treatment duration in group 1 (*P* > .05).

In multivariable linear regression analysis, considering only patients from group 1, there was no association between the duration of therapy or the cumulative dose of GCs with PWV, FMD, VAT, CIMT, serum IL-6, and IL-8 levels (*P* > .05).

Comparing all 47 patients (group 1 + group 2) with the controls, there were no statistically significant differences in BMI, HDL, TG, or HOMA-IR values (*P* > .05). Higher serum androgen levels were observed in patients (*P* < .05); however, no statistically significant differences were observed in PWV, FMD, VAT, VAT/SAT, or CIMT values or even in inflammatory cytokine levels (*P* > .05).

Another analysis was performed in group 1, which was divided into group 1A and group 1B. Fourteen patients were diagnosed during infancy due to precocious pubarche and received short-life GCs until the final height achievement, after which they received dexamethasone therapy (group 1A, n = 14). Group 1B comprised patients who received only dexamethasone (n = 14) during adulthood. Patients from group 1A were younger than those from group 1B; the mean age at the last evaluation was 28.6 ± 6.8 vs 37.5 ± 9.2 years, respectively (*P* = .014). Increased left CIMT was observed in group 1B in relation to group 1A (0.57 ± 0.09 vs 0.50 ± 0.05, respectively; *P* = .017). There were no statistically significant differences between the groups in relation to BMI, HOMA-IR, HDL, TG, IL-6, IL-8, and androgen levels and VAT, VAT/SAT, PWV, FMD, and CIMT values (*P* > .05).

We also performed an analysis comparing data from patients with obesity and normal BMI. As expected, patients with a BMI > 30 kg/m^2^ presented with higher VAT, HOMA-IR, TG, and serum leptin and lower HDL-c levels than those who were not obese. Interestingly, patients with obesity received a higher cumulative dose of cortisone acetate during growth periods (patients with obesity: 31,993, IQR: 28 265–41 357 mg/m^2^ vs patients with normal BMI: 20,715, IQR: 18 277–24 720 mg/m^2^; *P* = .03), but the dexamethasone doses used during adulthood did not differ (patients with obesity: 26,804, IQR: 17 938–52 112 mg/m^2^ vs patients with normal BMI: 31,144, IQR: 12 502–46 547 mg/m^2^, in hydrocortisone equivalence; *P* > .05). There were no statistically significant differences between the groups in relation to PWV, FMD, CIMT, serum IL-6, IL-8, and androgen levels (*P* > .05).

## Discussion

There is a discussion in the literature about whether patients with the NCAH present increased CVR, as observed in PCOS and patients with classical forms of CAH (4, 10). This is the first study that evaluated early markers of cardiovascular disease in a cohort comprising only patients with NCAH. Additionally, we also analyzed the influence of therapy on these markers; some patients were treated with low GC doses, while others were treated according to their symptoms.

We used validated methodologies for the early identification of CVR, such as the assessment of PWV, FMD, visceral fat, and CIMT, in this cohort of young adults with the nonclassical form, and we did not observe increased CVR. Similarly, we also did not observe significant differences in these parameters among patient groups according to therapy, ie, those receiving low GC doses and those treated symptomatically, in relation to matched controls.

Previous studies demonstrated increased serum C-reactive protein, PAI-1, and leptin levels in patients with classical and nonclassical forms in relation to controls (30, 31). These findings can be explained by the frequent use of supraphysiological GC doses to control significant hyperandrogenism (10). In the present study, we described higher serum IL-8 and IL-6 levels in patients from group 1, treated with dexamethasone. Interestingly, IL-6 levels were lower in group 2, treated with OCPs, than in the control group. The use of corticosteroids is known to reduce interleukin values, and we speculated that since we used low GC doses, it was not enough to result in the expected anti-inflammatory effect (32). Another hypothesis for these findings would be a protective role of estrogen from OCPs, which would have reduced serum interleukin levels in group 2, since previous studies have shown that interleukin levels are lower in premenopausal women than in menopausal women (33). Although group 1 presented higher serum interleukin levels, they were not correlated with changes in early markers of atherosclerosis. Considering that serum interleukin values did not correlate with the duration of GC therapy or with the cumulative dose of GCs, the question remains whether the difference between groups 1 and 2 may also have been influenced by an estrogenic protective effect in group 2. We emphasize that there are no previous studies evaluating serum IL-8 levels in patients with different clinical forms of CAH.

No significant differences in HOMA-IR, HDL, or TG levels between all patients and controls was observed. A previous study that evaluated 31 patients with NCAH treated with GCs showed lower HDL-c levels compared to controls and a higher waist-to-height ratio (31). Another Israeli study, which included 114 young patients with NCAH with a mean age of 17 ± 6.9 years at the last evaluation, observed that the proportion of overweight and obese patients in the sample was similar to that in the general population, and no alteration was observed in the lipid profile (23). Similar to our data, a meta-analysis that included 14 studies encompassing 437 patients with classical and nonclassical forms of CAH did not present significant differences in total cholesterol, low-density lipoprotein, HDL, or TG levels in CAH patients compared to controls (10).

In contrast to what was described for PCOS and patients with classical forms, we did not observe increased VAT and VAT/SAT in patients with the nonclassical form in relation to controls (4, 12, 13, 17, 30). A study including 28 adolescents with classical forms observed higher VAT and VAT/SAT in relation to matched controls and a positive correlation between VAT and HOMA IR, PAI-1, C-reactive protein, and leptin levels (30). We cannot rule out that these findings represent the need for higher GC doses to control androgen excess in patients with classical forms.

Interestingly, in the present study, when we subdivided patients from group 1 according to the period of life in which GC therapy was introduced, we did not observe a correlation between dose and duration of GC therapy with VAT or VAT/SAT. These data represent that long-term low GC doses in NCAH could not significantly influence visceral fat gain, at least in these young adult patients.

In a series of PCOS patients, it is known that hyperandrogenism may be related to increased insulin resistance (4, 17, 18). Insulin resistance was also described in patients with classical forms (10-12). A Brazilian study that evaluated insulin resistance using hyperinsulinemic-euglycemic clamp in 31 NCAH patients observed insulin resistance in this group (31). Although the 47 patients in our study had higher serum androgen levels than the controls, insulin resistance was not detected by HOMA-IR. We emphasized that there was no correlation between treatment duration or cumulative GC doses and HOMA-IR.

Despite the findings of serum IL-6 and IL-8 levels, we did not demonstrate any significant difference in CIMT between the patients with NCAH and controls. In the analysis of group 1, patients for whom treatment with GCs was initiated during childhood (subgroup 1A) had lower CIMT values than patients who received GCs only in adulthood (subgroup 1B), evidencing the influence of age on this parameter (34). In a recent meta-analysis (10), it was demonstrated that patients with classical and nonclassical forms, younger than 30 years, had higher CIMT compared to controls; however, in these studies, the treatment regimens were heterogeneous, with GC doses varying from 10 to 26.5 mg/m^2^/day in hydrocortisone equivalence. Higher CIMT values were also found in PCOS patients as compared to controls (17, 18). We cannot rule out that the suppression of hyperandrogenism in the early stages of development could corroborate the lower CIMT values in group 1A.

We did not observe a significant difference in FMD in patients with nonclassical forms in relation to controls—not even in the group that received GC therapy. Few studies in the literature have evaluated FMD in patients with the classical form, and lower values were observed in relation to controls (25, 26, 35). One of these studies also evaluated CIMT in 28 adolescents with a mean age of 14.8 ± 3.2 years and did not identify a significant difference in relation to matched controls. All these findings suggest that vascular dysfunction is observed in adolescents with classical forms when early detection methodologies are used (25). To corroborate this hypothesis, a Polish study evaluated 19 women with classical forms, with a mean age of 23.7 ± 3.8 years, and showed lower FMD than controls matched for age and BMI. However, the average dose of hydrocortisone used in this latter study was 20 mg/m^2^/day, aiming to normalize serum 17OHP values (26).

To our knowledge, for the first time, FMD was evaluated in patients with NCAH. Most likely, the lower severity of hyperandrogenism related to the nonclassical form, as well as the use of lower doses of GCs in our series, were not sufficient to increase CVR in this cohort of young adults.

We found no significant difference in PWV between patients and controls or according to therapy. In the literature, 1 study identified higher PWV values in 84 patients with classical and nonclassical forms in relation to controls (36); this study did not allow for the analysis of data exclusively from patients with the nonclassical form. It is noteworthy that our sample comprised young patients, and it has been demonstrated in the literature that changes in PWV have a positive correlation with age (27, 28). Although PWV did not differ significantly among the 3 groups, a weak correlation was observed between PWV and the duration of GC therapy, but no patient presented with a PWV value higher than 10 m/s. Although the mean duration of GC therapy in group 1 was 10.2 ± 7 years, 5 patients received treatment for more than 20 years, and PWV value ranged from 7.05 to 7.9 m/s.

In the analysis of patients with obesity vs patients with normal BMI, we observed insulin resistance and higher VAT values in the former group. Interestingly, patients with BMI >30 kg/m^2^ received higher cortisone acetate cumulative doses during growth periods than patients with normal BMI, and cumulative dexamethasone doses used during adulthood did not differ. Similar data were observed in classical CAH patients of our cohort. There is increased frequency of obesity as well as metabolic syndrome components during the transition period (9); after that, these frequencies remained stable during a mean follow-up of approximately 10 years. We speculated that the doses of cortisone acetate used in childhood to suppress the bone age advancement, rather than these low doses of dexamethasone in adult life, may be related to greater prevalence of obesity and adverse metabolic profile in adulthood.

One limitation of this study is that a convenience sample was used. We selected patients who were undergoing regular long-term follow-up in our service. Another limitation was the sample size of group 2, for which recruitment was hampered during the pandemic period. Another important aspect is that our sample comprised young adults, with a mean age of 33 years at the last evaluation.

In conclusion, the data presented in this series suggest that patients with NCAH do not present increased CVR, as assessed through specific radiological methodologies to identify precocious atherosclerosis. Considering the complexity of the inflammatory cascade, it is still too early to consider the isolated finding that increased serum IL-6 and IL-8 levels could result in increased inflammation in the long term.

## Data Availability

Original data generated and analyzed during this study are included in this published article or in the data repositories listed in References.
